# Successful Preoperative Transjugular Intrahepatic Portosystemic Shunt for Portal Decompression in Patients With Inflammatory Bowel Disease and Cirrhosis Requiring Surgical Intervention

**DOI:** 10.1093/crocol/otae037

**Published:** 2024-06-20

**Authors:** Christian Karime, Asrita Vattikonda, Jana G Hashash, Barry G Rosser, Amit Merchea, Luca Stocchi, Francis A Farraye

**Affiliations:** Department of Internal Medicine, Mayo Clinic, Jacksonville, FL, USA; Department of Internal Medicine, Mayo Clinic, Jacksonville, FL, USA; Inflammatory Bowel Disease Centre, Department of Gastroenterology and Hepatology, Mayo Clinic, Jacksonville, FL, USA; Department of Gastroenterology and Hepatology, Mayo Clinic, Jacksonville, FL, USA; Department of Colorectal Surgery, Mayo Clinic, Jacksonville, FL, USA; Department of Colorectal Surgery, Mayo Clinic, Jacksonville, FL, USA; Inflammatory Bowel Disease Centre, Department of Gastroenterology and Hepatology, Mayo Clinic, Jacksonville, FL, USA

**Keywords:** inflammatory bowel disease, cirrhosis, transjugular intrahepatic portosystemic shunt, colorectal surgery, operative outcomes

## Abstract

**Background:**

Colorectal surgery in patients with inflammatory bowel disease (IBD) and cirrhosis has increased morbidity, which may preclude surgery. Preoperative transjugular intrahepatic portosystemic shunt (TIPS) is postulated to reduce surgical risk. In this retrospective single-center study, we characterized perioperative outcomes in patients with IBD and cirrhosis who underwent preoperative TIPS.

**Methods:**

We identified patients with IBD and cirrhosis who had undergone preoperative TIPS for portal decompression between 2010 and 2023. All other indications for TIPS led to patient exclusion. Demographic and medical data were collected, including portal pressure measurements. Primary outcome of interest was perioperative outcomes.

**Results:**

Ten patients met the inclusion criteria. The most common surgical indications were dysplasia (50%) and refractory IBD (50%). TIPS was performed at a median of 47 days (IQR 34–80) before surgery, with reduction in portal pressures (22.5 vs. 18.5 mmHg, *P* < .01) and portosystemic gradient (12.5 vs. 5.5 mmHg, *P* < .01). Perioperative complications occurred in 80% of patients, including surgical site bleeding (30%), wound dehiscence (10%), systemic infection (30%), liver function elevation (50%), and coagulopathy (50%). No patients required re-operation, with median length of stay being 7 days (IQR 5.5–9.3). The 30-day readmission rate was 40%, most commonly for infection (75%), with 2 patients having intra-abdominal abscesses and 1 patient with concern for bowel ischemia. Ninety-day and one-year survival was 100% and 90%, respectively. Patients with primary sclerosing cholangitis (PSC)-cirrhosis were noted to have higher perioperative morbidity and a 30-day readmission rate.

**Conclusions:**

In patients with IBD and cirrhosis, preoperative TIPS facilitated successful surgical intervention despite heightened risk. Nevertheless, significant complications were noted, in particular for patients with PSC-cirrhosis.

## Graphical Abstract

**Graphical Abstract:**
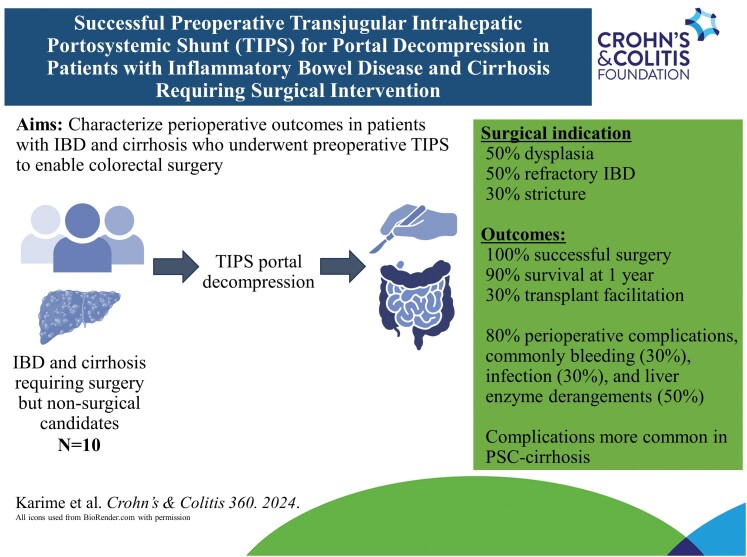

## Introduction

As part of the multidisciplinary longitudinal care of patients with inflammatory bowel disease (IBD), colorectal surgery may be required. This is most commonly for the management of medication-refractory disease, fibro-stenotic complications, or dysplasia. It is estimated that up to 30% of patients with IBD have abnormal liver tests and 5% develop cirrhosis, which may complicate planned colorectal surgery.^1^ The pathogenesis of liver dysfunction in patients with IBD is multifaceted, including a shared autoimmune background in primary sclerosing cholangitis (PSC), metabolic derangements in metabolic dysfunction-associated steatotic liver disease (MASLD), and IBD-associated medication toxicity.^1,2^

Colorectal surgery in patients with cirrhosis is associated with increased morbidity and mortality, with a further increase in post-operative complications in patients with portal hypertension.^3^ Transjugular intrahepatic portosystemic shunt (TIPS) is a minimally invasive procedure performed to reduce portal pressures and portal hypertension-associated complications. Given elevated surgical risk in patients with cirrhosis undergoing colorectal surgery, preoperative TIPS for portal decompression has been speculated to be a viable way to decrease perioperative complications and allow for surgical intervention in otherwise nonsurgical candidates.^4–6^ In this study, we aimed to characterize perioperative outcomes in patients with IBD and cirrhosis deemed nonsurgical candidates who underwent preoperative TIPS for portal decompression prior to colorectal surgery.

## Methods

### Patient Selection

**Figure 1.**
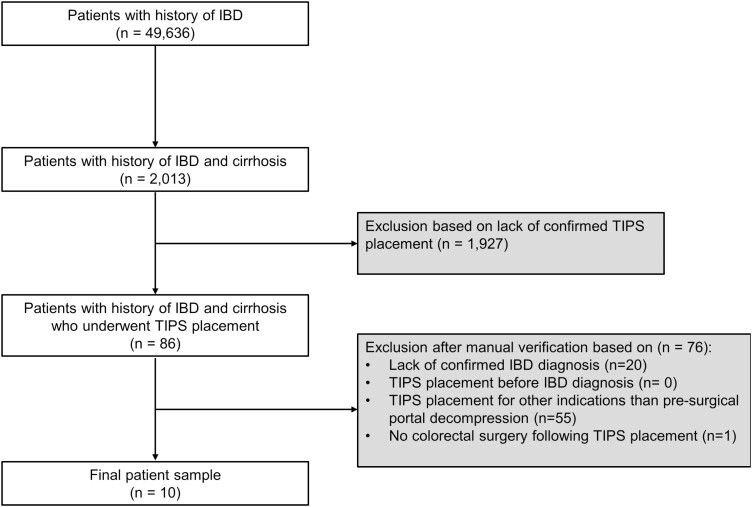
Patient selection diagram. IBD, inflammatory bowel disease; TIPS, transjugular intrahepatic portosystemic shunt.

We retrospectively identified patients with IBD and cirrhosis who had undergone TIPS placement between 2010 and 2023 prior to colorectal surgery (Figure 1). An initial cohort of 86 patients was identified using ICD-10 codes for IBD (K50.*, K51.*) and cirrhosis (K74.*) who had associated CPT codes for TIPS (37182, 37183). Verification of IBD diagnosis (confirmed by biopsy), cirrhosis (confirmed by cross-sectional imaging or biopsy), and TIPS placement (confirmed by procedural or cross-sectional imaging documentation) were performed. Chart review was completed to verify TIPS placement for a primary indication of portal decompression prior to planned colorectal surgery. Patients undergoing TIPS for other indications were excluded.

Additional exclusion criteria were: (1) age < 18 years, (2) TIPS prior to IBD diagnosis, (3) inability to verify TIPS placement, (4) inability to confirm diagnosis of IBD or cirrhosis. Following manual verification and exclusion, a total of 10 patients were identified. Institutional Review Board approval was obtained for the study.

### Data Collection

Demographic, clinical, and procedural data were collected. Demographic data included age at TIPS placement and gender. Clinical data included the Charlson Comorbidity Index, etiology of cirrhosis, model for end-stage liver disease-sodium (MELD-Na) score, and Child Pugh class. History of prior cirrhosis-related complications was also recorded, including ascites, splenomegaly, thrombocytopenia, hepatic encephalopathy, and esophageal varices with or without a history of bleeding. Procedural data included surgical indication (non-exclusive), pre- and post-TIPS portal pressure and portosystemic gradient measurement, time from TIPS placement to surgery, and type of surgery performed.

The primary outcomes of interest were procedural complications, divided into (1) TIPS-related complications and (2) perioperative complications. TIPS-related complications investigated included encephalopathy, volume overload, liver function elevation, hemorrhage, and thrombosis. Perioperative complications included intra- and postoperative blood transfusion requirement, postoperative bleeding (defined as provider documentation of surgical site bleeding), surgical site complications (including surgical site infection, wound dehiscence, anastomotic leak, and abscess formation), systemic complications (including systemic signs of infection, peritonitis, volume overload, respiratory failure, liver function test elevation, coagulopathy, electrolyte abnormality, and hypotension or shock). Systemic signs of infection were defined as hypotension, fever, and/or tachycardia with a suspected or known source of infection. Coagulopathy was defined as new international normalized ratio elevation ≥1.5, international normalized ratio elevation of ≥0.5 above baseline, or platelets ≤50 × 10^9^/L. Secondary outcomes included length of stay (LOS), mortality, need for reoperation, 30-day hospital readmission, and survival at 90 days and 1 year.

### Statistical Analysis

Descriptive statistics were utilized to summarize baseline characteristics and perioperative outcomes as medians with interquartile ranges (IQR) for continuous variables, and proportions for categorical variables. Wilcoxon Rank Sum and Fisher Exact tests were performed to compare pre- and post-TIPS portal pressure measurements. Analysis was performed using SPSS statistical software (version 25, IBM SPSS).

## Results

### Demographics and Surgical Indication

Following exclusion, 10 patients with IBD and cirrhosis were identified. Patient demographics and characteristics are detailed in Table 1. Most common etiology of cirrhosis was PSC (70%) followed by MASLD (30%). All patients had a history of decompensated cirrhosis, including esophageal varices (80%), ascites (70%), thrombocytopenia (70%), and a history of hepatic encephalopathy (40%). Most patients were Child Pugh class B (80%), with the remainder being Child Pugh class A (20%). The median Child Pugh score was 7.0 (IQR 6.8–8.0). The median MELD-Na score was 11.0 (IQR 8.8–13.0), with the highest MELD-Na score being 18. Both the Child Pugh score and MELD-NA score were calculated immediately prior to TIPS placement, with the time range from calculation to TIPS placement being 1–7 days.

**Table 1.** Baseline characteristics.

	Median (IQR) or fraction (%)
Variable	All patients *N* = 10
*Demographics*	
Age at time of TIPS placement (years)	59.7 (49.3–69.3)
Age at time of colorectal surgery (years)	59.9 (49.4–69.4)
Time between TIPS placement and surgery (days)	47.0 (34.0–80.0)
Male	6 (60.0%)
Caucasian	10 (100%)
*Comorbid conditions*	
Inflammatory bowel disease	10 (100%)
Crohn’s disease	3 (30.0%)
Ulcerative colitis	7 (70.0%)
Diabetes mellitus	3 (30.0%)
Chronic obstructive pulmonary disease	1 (10.0%)
Chronic kidney disease (stage 2 or higher)	3 (30.0%)
Congestive heart failure	1 (10.0%)
Transplant (solid organ or bone marrow)	0 (0%)
Charlson Comorbidity Index	6.0 (5.0–8.3)
Body mass index	28.1 (23.6–31.0)
Prior gastrointestinal surgery (not mutually exclusive)	3 (30.0%)
Total colectomy	1 (10.0%)
Partial colectomy	2 (20.0%)
Ostomy at time of TIPS placement	2 (20.0%)
*Characteristic of cirrhosis*	
Liver cirrhosis etiology	10 (100%)
Alcohol related	0 (0%)
MASLD	3 (30.0%)
Primary sclerosing cholangitis	7 (70.0%)
Prior history of decompensated cirrhosis	10 (100%)
Ascites	7 (70.0%)
Encephalopathy	4 (40.0%)
Thrombocytopenia (PLT < 150 000)	7 (70.0%)
Esophageal varices	8 (80.0%)
Varicealhemorrhage (any)	1 (10.0%)
Hemoglobin (g/dL)	11.20 (10.4–13.3)
Platelets (10^9^/L)	146.5 (105.5–212.5)
International normalized ratio	1.2 (1.1–1.4)
Albumin (g/dL)	3.1 (2.8–3.6)
Total bilirubin (mg/dL)	1.9 (0.7–3.1)
Direct bilirubin (mg/dL)	0.8 (0.4–1.5)
MELD-Na score	11.0 (8.8–13.0)
Child-Pugh score	7.0 (6.8–8.0)

MELD-Na score, Child-Pugh score, and baseline laboratory parameters were derived immediately prior to transjugular intrahepatic portosystemic shunt placement.

IQR, interquartile range; TIPS, transjugular intrahepatic portosystemic shunt; PSC, primary sclerosing cholangitis; MASLD, metabolic dysfunction-associated steatotic liver disease; MELD-Na, model for end-stage liver disease sodium.

The most common surgical indications were colonic dysplasia (50%), refractory IBD (50%), and luminal stenosis or stricture (30%; Table 2). All patients were initially deemed and documented as nonsurgical candidates by a multidisciplinary team which included colorectal surgeons due to significant operative risk related to portal hypertension and hemorrhage. Following multidisciplinary discussion and coordination, all patients underwent TIPS for a primary indication of portal decompression prior to a planned surgical procedure. TIPS was performed at a median of 47 days (IQR 34–80) prior to colorectal surgery, with the most commonly performed surgery being proctocolectomy (60%) and colectomy (20%; Table 2).

**Table 2.** Demographic and portal pressure measurements.

	Demographics								Portal pressure and surgical variables					
Patient number	Age at colorectal surgery (years)	Inflammatory bowel disease	Charlson comorbidity index	Etiology of cirrhosis	MELD-Na Score	Child Pugh class	Complication of cirrhosis	Surgery (surgical indication)	Portal pressures before TIPS (mmHg)	Portal pressures following TIPS (mmHg)	Portosystemic gradient before TIPS (mmHg)	Portosystemic gradient following TIPS (mmHg)	Time from TIPS to surgery (days)	Complication following TIPS
1	60	UC	6	PSC	10	B	Ascites Thrombocytopenia Splenomegaly	Colectomy with end ileostomy (refractory IBD)	23	20	10	4	168	None
2	66	UC	5	PSC	11	B	Ascites Hepatic encephalopathy Thrombocytopenia Esophageal varices Splenomegaly	IPAA reversal to diverting loop ileostomy (stricture)	24	19	20	13	49	None
3	45	UC	5	PSC	8	A	Ascites Hepatic encephalopathy Thrombocytopenia Esophageal varices Splenomegaly	Proctocolectomy with IPAA creation (dysplasia)	27	18	15	5	133	None
4	77	CD	8	MASLD	13	B	Hepatic encephalopathy Thrombocytopenia Esophageal varices	Colectomy with end ileostomy (refractory IBD, stricture)	17	11	13	3	34	Encephalopathy
5	58	CD	5	MASLD	9	B	Ascites Esophageal varices	Ileocecectomy with end ileostomy (refractory IBD, stricture)	27	24	12	6	29	None
6	68	CD	6	MASLD	11	B	Esophageal varices Splenomegaly	Proctocolectomy with end ileostomy (refractory IBD)	18	10	16	6	63	None
7	70	UC	12	PSC	12	B	Ascites Thrombocytopenia Esophageal varices	Proctocolectomy with end ileostomy (dysplasia)	18	11	8	2	43	Volume overload
8	49	UC	9	PSC	13	B	Ascites Hepatic encephalopathy Thrombocytopenia Esophageal varices Splenomegaly	Proctocolectomy with IPAA (dysplasia)	20	16	7	6	45	None
9	53	UC	4	PSC	8	A	Ascites Thrombocytopenia Splenomegaly	Proctocolectomy with end ileostomy (dysplasia)	22	19	8	4	34	Volume overload
10	49	UC	8	PSC	18	B	Esophageal varices with variceal bleed Splenomegaly	Proctocolectomy with end ileostomy (dysplasia, refractory IBD)	30	25	14	8	49	None
Median (IQR) or Fraction (%)	59.9 (49.4–69.4)	70% UC 30% CD	6.0 (5.0–8.3)	70% PSC 30% MASLD	11.0 (8.8–13.0)	A: 20% B: 80%	Ascites: 70% Hepatic encephalopathy: 40% Thrombocytopenia: 70% Esophageal varices: 80% Splenomegaly: 70%	30% colectomy 50% proctocolectomy 20% other	22.5 (18.0–27.0)	18.5 (11.0–21.0)	12.5 (8.0–15.3)	5.5 (3.8–7.3)	47.0 (34.0–80.0)	30%

IBD, Inflammatory bowel disease; IPAA, ileal pouch-anal anastomosis; IQR, interquartile range; CD, Crohn’s disease; UC, Ulcerative colitis; TIPS, Transjugular intrahepatic portosystemic shunt; PSC, Primary sclerosing cholangitis; MASLD, Metabolic dysfunction-associated steatotic liver disease.

### Procedural Outcomes and Complications

#### TIPS related outcomes

Placement of TIPS was associated with a significant reduction in portal pressure (22.5 vs. 18.5 mmHg, *P* < .01) and portosystemic gradient (12.5 vs. 5.5 mmHg, *P* < .01) in all patients. TIPS-related complications occurred in 3 patients (30%), most commonly volume overload (20%), and hepatic encephalopathy (10%).

#### Perioperative outcomes

In terms of perioperative complications, 80% of patients developed either intra- or post-operative complications (Table 3). Intraoperatively, 40% of patients required blood transfusion (range: 1–4 units of packed red blood cells). Postoperatively 30% of patients had self-resolving surgical site bleeding and 10% had superficial wound dehiscence, although none required post-operative blood transfusion or re-operation. Superficial wound dehiscence was managed conservatively in patient #9. No other surgical site complications were noted during the index hospitalization, including no anastomotic leak, abscess formation, or other surgical site infection.

**Table 3.** Perioperative complications, 30-day readmission, and survival.

	Perioperative complications								30-day post-operative complications			Survival	
Patient	Intra-operative blood transfusion	Post-operative Bleeding	Post-operative blood transfusion	Surgical site complications	Systemic complication	Liver function elevation	Coagulopathy	Venous thromboembolism (including portal vein)	Hospital readmission	Complications	Re-operation needed	90-day survival	1-year survival
1	Yes	No	No	None	None	No	No	No	No	—	—	Yes	Yes
2	No	No	No	None	Systemic infection	No	Yes	No	Yes	Systemic infection Intra-abdominal abscess	No	Yes	Yes
3	Yes	Yes	No	None	None	Yes	No	No	Yes	Systemic infection Intra-abdominal abscess	No	Yes	Yes
4	No	No	No	None	None	No	No	No	No	—	—	Yes	Yes
5	Yes	Yes	No	None	None	Yes	Yes	No	No	—	—	Yes	Yes
6	No	No	No	None	None	No	No	No	No	—	—	Yes	Yes
7	Yes	No	No	None	Systemic infection Heart failure exacerbation Volume overload	Yes	Yes	No	Yes	Electrolyte abnormality Volume overload	No	Yes	No
8	No	Yes	No	None	None	No	Yes	No	No	—	—	Yes	Yes
9	No	No	No	Wound dehiscence (superficial)	Systemic infection Peritonitis Encephalopathy Respiratory failure Shock	Yes	Yes	No	Yes	Systemic infection Electrolyte abnormality Bowel ischemia Wound dehiscence	Yes	Yes	Yes
10	No	No	No	None	None	Yes	No	No	No	—	—	Yes	Yes
Median (IQR) or Fraction (%)	40%	30%	0%	10%	30%	50%	50%	0%	40%	40%	10%	100%	90%

In terms of perioperative complications, surgical site complications recorded included surgical site infection, wound dehiscence, anastomotic leak, abscess formation, and bleeding. Systemic complications recorded included systemic signs of infection, peritonitis, volume overload, respiratory failure, liver function elevation, coagulopathy, electrolyte abnormality, and hypotension or shock. Systemic signs of infection were defined as hypotension, fever, and/or tachycardia with a suspected or known source of infection.

Systemic complications were noted in 30% of patients, all of which had a history of PSC-derived cirrhosis. Systemic signs of infection were the most common (30%) systemic complication, with a urinary source suspected in patients #2 and #7 and secondary bacterial peritonitis occurring in patient #9. Patient #7 experienced heart failure exacerbation in the setting of volume overload, which was resolved with intravenous diuresis. Patient #9 experienced signs of peritonitis and encephalopathy with hemodynamic and respiratory compromise. The patient required intensive care unit management with intravenous antibiotics, temporary renal replacement therapy, and mechanical ventilation with subsequent recovery. The presumed cause of decompensation was thought to be secondary bacterial peritonitis following surgical intervention. Other systemic complications included transient liver function test elevation (50%) and coagulopathy (50%). Median hospital LOS following surgery was 7 days (IQR 5.5–9.3), with no patient requiring re-operation and without in-hospital mortality.

The 30-day readmission rate was noted to be 40%. All readmitted patients had a history of PSC-cirrhosis. The most common cause for readmission was signs of systemic infection (75%) and electrolyte abnormalities (50%). During readmission, 2 patients were found to have new intra-abdominal abscess formation (patient #2 & 3). Both patients were treated with antibiotics without the need for reoperation or drainage. On readmission, patient #9 was found to have superficial wound dehiscence with cross-sectional imaging noting diffuse small bowel thickening and a small area of intestinal pneumatosis. Given the concern for bowel ischemia with perforation, the patient underwent explorative laparotomy. Wound closure of dehiscence was performed with subsequent continuous negative pressure wound therapy. No bowel perforation was identified during laparotomy. The median readmission hospital LOS was 8.5 days (IQR 2.3–16.3) without mortality.

On longitudinal follow-up, the 90-day and 1-year survival was found to be 100% and 90%, respectively. Patient #7 unfortunately was readmitted due to electrolyte disturbances in the setting of hepatorenal syndrome. Over the course of the hospitalization, the patient developed multiorgan failure. Following multidisciplinary patient-centered goals of care discussion, the patient and family decided to pursue home hospice and the patient regrettably passed away. After colorectal surgery, 2 patients (#2 & #8) were listed for liver transplantation. The patients were listed at 7 and 8 months after colorectal surgery, respectively, with patient #8 being transplanted successfully 5 months after listing. Patient #10 was listed for a liver transplant 6 months prior to TIPS-enabled colorectal surgery. Two months following surgical intervention, patient #10 successfully underwent liver transplantation.

## Discussion

Given the elevated operative risk in patients with cirrhosis, preoperative TIPS for portal decompression has been speculated as a viable way to decrease operative complications and allow for surgical intervention in otherwise nonsurgical candidates.^4–6^ However, prior results have not been consistent, and data is scarce in patients with IBD and cirrhosis with the largest study limited to 9 patients.^7^ As such, we aimed to retrospectively characterize the perioperative outcomes in patients with IBD and advanced cirrhosis who underwent preoperative TIPS placement for portal decompression prior to surgical intervention. In the 10 patients identified, TIPS successfully lead to portal decompression in all patients with relatively few TIPS-related complications. In terms of perioperative outcomes, all patients were able to undergo the required surgical intervention, most commonly colectomy or proctocolectomy. Given that the most common indications for surgery were dysplasia and refractory/complicated IBD, TIPS decompression allowed for surgical intervention and prevention of potentially life-changing and life-threatening complications such as malignancy progression and IBD disease morbidity in a patient population who would otherwise not have been able to undergo surgical intervention. Additionally, colorectal surgery enabled liver transplant listing for 2 patients who may not otherwise have been viable candidates. Nevertheless, the rate of perioperative complications were noted to be high, congruent with prior research in patients with IBD and cirrhosis.^7–9^

Compared to patients with MASLD-derived cirrhosis, our study noted that patients with PSC-cirrhosis had higher perioperative morbidity, with more frequent intraoperative blood transfusions (42.9% vs. 33.4%), a higher rate of systemic post-operative complications (42.9% vs. 0%), and a higher 30-day readmission rate (57.1% vs. 0%). This finding was noted despite similar Child-Pugh class (100% vs. 71.4% class B) and MELD-Na score (11.0 vs. 11.4), with MASLD patients being on average older at the time of surgery compared to patients with PSC (67.6 vs. 56.0 years). However, it should be noted that the mean time between TIPS placement and surgery was shorter in patients with MASLD-cirrhosis compared to PSC-cirrhosis (42.0 vs. 74.4 days), with a resultant lower portal pressure (15.0 vs. 18.3 mmHg) and portosystemic gradient (5.0 vs. 6.0 mmHg). As such, it could be hypothesized that the difference in outcomes could be related to a shorter TIPS-to-surgery interval and greater portal decompression. However, currently, data is limited regarding optimal colorectal surgical timing following TIPS. Although research suggests that portal pressures are reduced immediately following TIPS and so risk reduction for perioperative complications may be reduced early, a new hemodynamic equilibrium and its clinical effects may take time.^10^ Future research may benefit from further exploration as to the association between PSC-cirrhosis and higher perioperative morbidity, and if patients with PSC-cirrhosis may be less conducive candidates for TIPS portal decompression. Future research would also benefit from exploring if the association with higher morbidity is independent or dependent on the TIPS-to-surgery interval or post-TIPS portal pressure measurements.

There are several limitations of the current study which should be noted. Firstly, the study is limited by its single-center design, rarity of disease condition, and stringent inclusion criteria that limited patient selection to only those who underwent TIPS for a primary indication of portal decompression prior to planned surgery. As noted in Figure 1, out of more than 2000 patients identified with IBD and cirrhosis, only 10 patients met the inclusion criteria. Nevertheless, the stringent inclusion criteria are also a strength of the study, allowing focus on only those patients whose operative risk prevented surgery without the use of TIPS. Furthermore, our study is limited by a lack of a comparison group, limiting inferences to patients whose cirrhosis was severe enough to be deemed nonsurgical candidates without portal decompression with TIPS. Although a comparison group may have allowed for expanded analysis, it was felt that retrospectively comparing outcomes in patients with IBD and cirrhosis who did versus did not undergo preoperative TIPS would have been problematic due to differences in severity of disease requiring TIPS decompression in 1 group but not the other. Indeed, this very issue was encountered by Kochhar et al. in a prior retrospective case–control series in a similar patient population with IBD and PSC requiring colorectal surgery, limiting the ability to draw conclusions as patients who underwent TIPS were noted to have more severe PSC and liver dysfunction.^7^ As such, whilst limited conclusions can be drawn regarding who may or may not benefit from TIPS decompression prior to colorectal surgery, our study provides insight and experience regarding a possible avenue of approach for provider care teams who are at an impasse in next care approaches for patients requiring surgical intervention but whose operative risk are great enough that surgery cannot be safely performed. Lastly, given its retrospective nature, limited conclusions can be made as to the timing of TIPS prior to surgery and its relation to perioperative complications, with further large-scale studies needed to determine optimal surgical timing following TIPS placement.

In conclusion, in our series of patients with IBD and cirrhosis who were deemed nonsurgical candidates, preoperative TIPS led to portal decompression and facilitated successful surgical intervention addressing IBD-related complications such as dysplasia and refractory disease despite heightened risk. Nevertheless, significant peri- and post-operative complications were noted, in particular for patients with PSC cirrhosis. Although the utility of preoperative TIPS to decrease perioperative complications is difficult to discern, TIPS decompression may be a potentially viable alternative in patients with cirrhosis who require colorectal surgery but are deemed nonsurgical candidates due to heightened operative risk.

## Data Availability

Data not publically available. Data available on reasonable request to authors.
